# Analysis of Stress Factors for Female Professors at Online Universities

**DOI:** 10.3390/ijerph17082958

**Published:** 2020-04-24

**Authors:** Marialuz Arántzazu García-González, Fermín Torrano, Guillermo García-González

**Affiliations:** Escuela Superior de Ingeniería y Tecnología, UNIR-Universidad Internacional de la Rioja, Av. de la Paz, 137, 26006 Logroño, Spain; marialuz.arantzazu@unir.net (M.A.G.-G.); guillermo.garcia@unir.net (G.G.-G.)

**Keywords:** telecommuting, technostress, higher education teachers, psychosocial risk factors, Delphi technique, women

## Abstract

The aim of this paper is to analyze the primary stress factors female professors at online universities are exposed to. The technique used for the prospective and exploratory analysis was the Delphi method. Two rounds of consultations were done with fourteen judges with broad experience in health and safety at work and university teaching who reached a consensus of opinion regarding a list of nine psychosocial risk factors. Among the most important risk factors, mental overload, time pressure, the lack of a schedule, and emotional exhaustion were highlighted. These risk factors are related to the usage and expansion of information and communication technology (ICT) and to the university system itself, which requires initiating more research in the future in order to develop the intervention programs needed to fortify the health of the affected teachers and protect them from stress and other psychosocial risks.

## 1. Introduction

The current status of telework, as defined by Eurofound and the International Labour Organization [1] is using information and communication technology (ICT; smartphones, tablets, laptops, ultra-books) for working outside the office. ICT is growing significantly and developing rapidly and accounts for an increasingly larger part of the world of work due to its potential benefits (autonomy, flexibility, transport, and cost savings, and even as a means for epidemiological containment for certain illnesses, such as coronavirus Covid-19) [2,3,4,5]. The Third European Survey of Enterprises on New and Emerging Risks (ESENER 3) [6] highlights that one of the earliest and greatest impacts of digitalization is increased flexibility for workers in terms of workspace and time, which points to a significant immediate and future growth in this kind of working.

Effective integration of ICT into the workplace has brought about substantial changes to the world of work that crosses limits in space and time, kinds of supervision, the work–life balance, and how the concept of work itself may be understood [7,8,9]. On top of that, there are other political, social and economic changes that have given rise to emerging psychosocial risks, such as technostress, that have negative effects on people and may cause higher levels of exhaustion and emotional stress [6,10].

University teaching is one of the contexts where digitalization has made a significant impact. It has led to the emergence of a new modality of teaching online that is expanding rapidly [11,12,13]. At the same time, a significant part of European telecommuters are categorized as so-called knowledge workers, who are highly qualified individuals who work primarily from home. Virtual university professors can be included in that category [1]. On the other hand, in this new scenario the use of virtual environments has been normalized without having evaluated the vulnerability of the people who work in them to negative effects derived from technological innovations, such as higher levels of stress and physical (musculoskeletal disorders) and psychological disorders (burnout, anxiety, and depression), with the consequent increase in work absenteeism [14,15,16,17,18].

The very few studies about this show the presence of significant psychological stress factors linked to using ICT and the organizational dynamics specific to this kind of virtual teaching. For example, isolation derived from a lack of face-to-face contact is one of the psychosocial risk factors that is frequently reported and consubstantial with online university teaching [19,20,21,22,23]. This is not only because it makes formal and informal communication within the organization more difficult, but also seems to limit the possibilities for organizational promotion and support [24], which may give rise to disappointment, distress, and alienation [25]. Alongside that, high workloads and the blurred edges between work and family spaces are other aspects that are perceived negatively by workers.

Excessive workload is related to the different roles university professors must fill beyond their teaching duties, such as administrative tasks, research, or organizing seminars [26,27,28]. It is likewise associated with work being concentrated in spikes, which is an intrinsic characteristic of online teaching. Insofar, as the edges between work and family life are blurred, on the one hand, it makes it harder to leave work behind and, on the other hand, it is related to an overload due to tasks overlapping in both spheres, which consequently becomes a real obstacle that needs to be coped with [29,30,31].

Furthermore, an important part of this research shows a higher vulnerability among female university professors in terms of experiences with and the consequences of stress [32,33,34]. Specifically, the prior studies show unfavorable differences for women in several factors, like mental overload and difficulties with work–life balance [35,36,37]. These differences are associated with a variety of factors linked to the modality of online work and to responsibilities distributed according to gender roles. Working from home increases the number of hours of work [7] and blurs the boundaries between the family and the workplace. In the case of women, schedule is restructured according to family needs, which can be explained from the perspective of traditional gender roles. According to this perspective, women are, mostly, responsible for family duties, thereby generating an overload of work in both fields [38,39]. Furthermore, women must deal with high levels of stress in both domains, which frequently leads to considering the option to stop working or professional resignation [28].

In light of the above, it should be a high priority to undertake research in the field of online university teaching that explores and scrutinizes the psychosocial aspects from a woman’s perspective. This study emerged in response to that need with the goal of identifying the psychosocial risk factors that affect professors who work at virtual universities. The purpose of it is to outline an initial approach to this topic, in line with the European Strategic Framework on Health and Safety at Work 2014–2020 report where the need to evaluate the psychosocial risk factors present in the workplace is underlined, specifically for women, because they may be one of the most vulnerable demographics [40].

## 2. Materials and Methods 

The Delphi technique, which is widely used in the field of social sciences and education, was chosen as the research technique [41]. With that prospective and exploratory technique, a group consensus about a topic based on expert opinion can be obtained through two or more rounds of consultations [42].

### 2.1. Participants

In the selection of the panel members, the expert was taken as the person who could provide information or opinions due to their involvement with the subject matter of the study. An evaluation of the judgers or experts was done by selecting those professionals who met one of the two following criteria: (a) experience at a theoretical level in occupational risks, and specifically, in psychosocial risks, evaluating research work and publications in scientific journals; and (b) having worked from a practical point of view in the evaluation, training, and prevention of occupational risks, specifically of a psychosocial nature. Other aspects were taken into account including work history, specifically, years of experience in the field of health and safety at work and online university teaching. Finally, the degree of motivation was also taken into account.

Initially, 25 experts from different areas of Spain who met one of the two requirements mentioned above were contacted by e-mail, and were informed about the aims of the study, the procedure and the tasks to be carried out, and were asked to collaborate by providing more experts who could participate. The research group finally contacted a total of 29 experts. Of this initial group, 15 experts responded to the request, which was finally reduced to 14, as a loss occurred between the first and second consultation rounds. The remaining 14 were the experts who made up the final panel of judges. 

The size of the panel made it possible to obtain sufficient and reliable information and anticipate that potential losses during the data collection process could occur and that it would not affect the quality of the findings. In those regards, Delbecq, Van de Ven, and Gustafson [43] highlight that 10–20 experts are enough if the sample is homogeneous in terms of educational level and degree, as shown in the panel of the present study.

In terms of distribution, seven respondents were senior occupational health and safety technicians at private companies, and the remaining seven were online university professors, three of whom also work as Occupational Health and Safety Technicians (OHST). The average experience of the expert group was around 10 years, with a minimum of five years in one case up to a maximum of 15, which ensured broad experience and knowledge in the field of online university teaching and workplace health and safety. Table 1 provides a more detailed description of the socio-demographic characteristics of the experts on the panel.

### 2.2. Procedure

**Figure 1:**
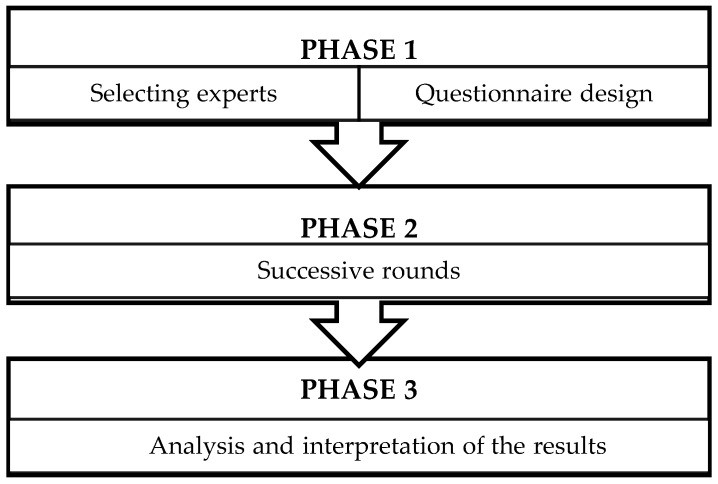
Delphi Technique: phases and procedure.

The procedure used is shown in Figure 1.

Two main tasks were done in the first phase, such that the experts were selected and the questionnaire used to start the first round of consultations was designed. 

Three open questions were devised for the questionnaire design through which the experts, in a brainstorming style, could freely indicate every ergonomic, psychosocial, and health risk factor they viewed as being related to the profession of online university teaching. The experts were asked to briefly justify the reasons for their choice about each of the three questions, which facilitated the analysis and interpretation of the information collected.

The validity of the content of the questionnaire was analyzed using the individual aggregate method [44], carried out by six external researchers from three different Spanish universities, who pointed out different improvements in the formulation of the questions.

The questions were as follows:

- What do you think are the risk factors on physical health among women who use ICTs in the online university teaching environment?
- What do you think are the psychosocial risk factors affecting women who use ICTs in the online university teaching environment?
- What do you think are the risk factors derived from the ergonomics and safety conditions for women using ICTs in the online university teaching environment?

The questionnaire was sent to the panel of experts by email. A letter was attached to the email reminding the experts about the goals of the Delphi technique, the procedures, and basic instructions for responding. In the first round, each expert was approached individually and not as a group or panel of experts.

Every response to the open questions from the panel was analyzed and categorized after the first round was completed. A list was then selected with 41 factors or categories that had a percentage of assignation equal to or greater than 50%. This was used to create a second structured questionnaire, on which information was provided to the evaluators about the percentage obtained for each factor (controlled feedback).

Every expert had to make two kinds of judgements based on that information:

- Whether they agreed with the percentage of assignment for all of the 41 items chosen. Agreement, expressed with a YES, indicated the expert believed that the item was a risk factor for the profession of online university professor. On the contrary, disagreement, expressed with a NO, meant that they did not believe the item was a risk factor for that job.
- If the response was affirmative the expert had to rate the significance of each item on a Likert-type scale, from 1 (not very important) to 10 (very important). If the answer was negative in the previous box, they did not give a rating.

The analysis and interpretation of the data collected was done, where a second quantitative threshold criteria was applied with the goal of finding out which items the panel reached a consensus about. At no time during the process did the members know the individual and personal response of the other members of the panel, in accordance with the principle of anonymity of the technique was used. At no time during the process did the members know the individuals or personnel. Lastly, the final report was written and submitted to the members of the panel.

The ethical principles underlying the research studies have been strictly observed. In accordance with the ethical standards included in the 1979 Belmont Report for the protection of human subjects participating in research, there are three general ethical principles that should guide any research: autonomy, beneficence, and justice.

Threshold criteria was applied with the goal of finding out which items the panel reached a consensus about: a first selection of those factors valued as important by the experts, considering the percentage of experts who point out each category (equal or above 60%); the mean and the standard deviation for each factor or category; and, in a follow-up, the analysis of the coefficient of variation obtained for each item.

The reliability of the results has been determined by considering the stability of the experts in both rounds (14 of the 15 members who were selected were still there), the time between rounds (the answers were collected in approximately two and a half months), and the involvement and quality of the qualitative contributions of the experts (highly positive).

Regarding the validity of the results, the research group considered, in coherence with the objectives of the research, that the results and their analyses would become the starting point for future qualitative research, which will delve into these factors found, as seen from the perspective and viewpoint of the online professors themselves. Furthermore, it was assumed that the results would be equal to or superior in quality to those obtained with other more expensive techniques.

### 2.3. Data Analysis

A number of descriptive analyses, calculating percentages, frequency tables, and centralization (mean), dispersion (standard deviation), and y parameters (coefficient of variation) statistics were used for analyzing the data. The IBM SPSS Statistics 22 (IBM Corporation, Armonk, New York, NY, USA) software was used for analysis. 

With the analysis in both rounds, the intention was to discover the items for which a consensus was reached Consensus implies obtaining a degree of convergence between individual judgements and it is reached when the opinions expressed by the experts reach an acceptable level of nearness or proximity. In the first round, an assignation percentage of judgements of 50% or higher was set as the quantitative threshold criteria, while in the second round the agreement percentage among respondents was increased to 60% or higher. Other results were also analyzed in the second round, such as the importance given to each factor and its relevance, which was determined by reaching a mean equal to or higher than six points and a standard deviation lower than two points.

## 3. Results

As indicated above, the expert consultation was done in two successive rounds, which was when a sufficient consensus between judgements was obtained in regard to the risk factors present in the profession of online university teaching from a gender perspective.

In the first round, based on the information collected in three open questions, a list of all the risk factors enumerated by the experts was made. There were 159 items on the list referring to different physical, ergonomic, psychosocial, and health related factors. Out of the total elements enumerated, the items that were indicated as primary risk factors by a greater number of panel members were selected.

The threshold criteria of the first round (assignation percentage equal to or greater than 50%) was applied to select 12 items, specifically: inadequate lighting (71.60%); inadequate temperature (71.42%); noise level, static posture, and technostress (64.29% each); use of Visual Display Terminal (VDT), neck pain, emotional overload, back pain, and visual fatigue (57.14% each); impacts with objects (50.61%); and falls on a single level or to a lower level (50%). Likewise, after those 12 items were selected, consistent with the specialized literature [45], the decision was made to select items with a higher figure from among the factors with an assignation percentage below 50%. The 41 items resulting from the first round of analysis that were part of the second questionnaire are shown in Table 2.

In the second round of consultations, and with the data obtained from using a second structured questionnaire, the intention was to advance to the final list of factors for which a consensus was reached. Table 3 shows the frequency and percentage of agreement between the panel members for all 41 items, as well as the mean and standard deviation for each one with an interval of 1–10 over the importance and relevance of the risk factor and the coefficient of variation (CV) obtained.

As can be seen, 28 items obtained percentages of agreement between panel members equal to or greater than 60%, which can therefore be considered risk factors. In relation to their importance, as can be observed, they also have a high mean score, above six points, and low variability, with a standard deviation below two points. Only the items (difficulty with work–life balance and level of humidity) that had a higher than established agreement percentage (85.7% and 64.3%, respectively) obtained a degree of importance lower than six and were consequently excluded because they did not meet the required consensus requirement. As shown in Table 3, in the 26 pre-selected items, an important consensus was been obtained among experts due to low or very low coefficients of variation. In this sense, the guidelines of English and Kernan [46] were followed, whereby a coefficient above 0.80 indicates the need for modifications or a new consultation. Therefore, in the case of this study and in light of the data obtained, the process was considered to be complete.

The list of risk factors and risks for which consensus was obtained in the second round is shown in Table 4.

Of the 26 risk factors about which consensus was reached by the experts, a significant number fall in the psychosocial category (*n* = 12) of high incidence and significance for teaching activities. Three of those factors (burnout, technostress and stress) were categorized as psychosocial risks. This is evident, for example, in the ESENER 3 survey [6]. Consequently, they were ultimately excluded from the final list of psychosocial risk factors, which was reduced to nine (see Table 5).

## 4. Discussion

The goal of this study was to examine the primary stress factors that women working as online university professors are exposed to that may lead to elevated levels of stress or burnout. The results obtained through two rounds of consultations with expert panel members shows, through consensus, the presence of nine psychosocial risk factors. Those items are intrinsically linked to new ways of organizing work derived from advances in ICT that have made it possible for individuals to work partially or wholly from home using ICT and that have affected the dynamics of online university teaching.

Regarding the quantity and complexity of information and demands on attention, the expert panel coincides in the cognitive dimensions of the tasks typically done by online university professors; specifically, in regard to the level of cognitive demands not just qualitatively (situations that require high intellectual effort), but also quantitatively (derived from situations with a high work load that require sustained attention over time). The continuous presence of the labor demands related to developing mental fatigue [17] have serious consequences for the cognitive and emotional processes of individuals, whose efficiency and performance are notably reduced [34,47]. At the same time, this aspect is related not only to the tasks, but also to the main tool of online teaching, namely the effective management of ICTs, which involves permanent training and updating of knowledge, skills, and abilities in the face of the rapid and vertiginous quantitative and qualitative changes in the technological field, in an effort to avoid techno-stress [48]. On the other hand, in the current global pandemic of Covid-19, the importance of this training and updating of online teaching strategies and tools for all educators can be appreciated at the different educational levels, in light of the mandatory cancellation of face-to-face teaching and the difficulties that this is causing for all teachers.

Another stressor the panel members concurred on regards the pace of work and is centered on time pressure linked with the deadline characteristics of online university teaching. This kind of teaching, as mentioned above, entails significant cognitive and motivational demands with strict deadlines. Time pressure occurs when the time available to complete tasks is less than the time needed. That situation leads to an increase in working hours beyond contractual schedules and reducing personal and family time to meet job demands [34,49,50].

Insofar as the lack of a schedule, it is seen as an aspect tied to the time dynamics of online teaching and teaching in general, where the volume of work spikes drastically in certain situations, such as exams, when the workload is doubled but there is no increase in the time allotted to tackle it. That mental overload affects female professors’ motivation and makes a work–life balance more difficult [51]. This aspect takes on particular importance in the case of female professors with school age children, for whom workload spikes bring about a role conflict derived from the demands of both spheres and they feel obligated to extend their working hours into the night and weekend [34].

These two risk factors appear to make it difficult to reconcile work and family and are major sources of stress, as women workers value the effectiveness and benefit of working from home insofar as it reduces work–family conflict compared to men who work from home for better job performance [52] Female university professors have greater control over the demands of work and the needs arising from family and domestic care [53]. This is closely related to the tasks assigned to them in accordance with traditional gender roles, which underline their greater responsibility in the family [2]. Most domestic tasks are “low-control-schedule” [54], which means they must be carried out despite interference, so that female teachers try to reorganize their activity around satisfying these family demands, and are forced to increase their schedules thanks to the possibilities of ICTs or, alternatively, reduce family time [38], as noted above. This eventually leads to work overload and high levels of stress, which are enhanced by the blurred boundaries between work and family, resulting from online education, work spikes, and time pressure linked to short and strict deadlines, all of which make it more difficult to reconcile the two fields.

Role ambiguity is the sum of personal and external expectations about the behavior appropriate for a role, regardless of the person who occupies it [55]. In research, it is related to different negative effects, such as higher workplace tension, depression, fatigue, and low self-esteem [56]. The fact that the panel of experts designated that factor as a potential stressor indicates the difficulties female online university professors face with having precise and detailed knowledge about the behavior, responsibilities, and expectations associated with their role because of a lack of direct personal communication with their supervisor. Consequently, it seems important to discover to what extent workers perceive the definition of their role, the goals they must meet, the activities they must do, and how those activities are scheduled, as well as the evaluation system. In addition, it would be interesting to explore the behavior styles associated with their role, which is a question that should be the subject of future research.

Emotional exhaustion is another psychosocial risk factor highlighted by the panel of experts. For female professors, this aspect is materialized in classes and contact with students in order to influence their attitudes and behavior. An emotional dissonance is sometimes created between the emotional expression stipulated by the organization and workers’ experiences. When this happens, these people should be provided with personal resources and emotional skills to help them lower the level of tension caused by the disparity [57,58]. Baker, Demerouti, and Schaufeli [59] also point out that emotional demands are one of the most important health predictors among female online workers.

The lack of autonomy is another potential stressor the experts agreed upon. Specifically, it is viewed as a possible hazard because of the excessively rigid scheduling in online teaching and the bureaucratization of the evaluation systems that causes the pace of work to be imposed by the educational system instead of workers. On the other hand, the lack of prior training in using ICT also (negatively) influences the perception of control and autonomy. In any event, this is considered to be a matter that later studies should explore more deeply due to that factor, alongside flexibility, being the aspect most highly valued by telecommuters [60]. 

Another psychosocial risk factor was associated with not having healthy habits, such as spending too much time in a sedentary position in front of a computer without taking enough breaks, which can cause the onset of musculoskeletal disorders and visual fatigue [17,61]. Also associated with that is using non-ergonomically designed workspaces and furniture, especially when working from home. 

The last psychosocial risk factor consensus was reached about is the lack of social recognition related to the devaluation of the online work model, primarily among university professors who teach face-to-face classes and who, according to Kurland and Bailey [62], need to change the existing telecommuting culture and the terms it is defined with. Those researchers warn of the risks derived from turning telecommuting into an abstract activity mediated by a computer; in other words, turning it into a dehumanized activity that can eventually cause a significant degradation of social skills for interacting adaptively with others. Since it is usually women who tend to take advantage of teleworking [1], this lack of social recognition mainly affects them and is linked to the lack of approval of non-domestic work for which they are considered to be the main person responsible. In this sense, Castaño [63] states that teleworking may contribute to making women’s work invisible, as it is now completely developed in the personal area. Finally, and in agreement with Pocock [64], it is understood that identifying the reasons why these psychosocial risk factors and the consequences associated with mimicry affect women online teachers more than men (generating higher levels of stress), requires a global approach focused on exploring the interrelations between multiple work, technological, social, and individual factors, which will be the subject of further research.

## 5. Conclusions

Even though telecommuting in the field of online university teaching was originally set forth as a mode of working that facilitates autonomy and better a work–life balance because of the flexibility with hours and location it advocates, our results show the presence of several stressors or psychosocial risk factors such as mental overload, time pressure, lack of a fixed schedule, and emotional exhaustion may lead to the appearance of several psychosocial risks including stress, burnout, and difficulty with the work–life balance.

As far as limitations of the study, in regard to the first round of consultation the panel experts were not given an unambiguous definition of the concept of teleworking, which would have been a baseline in terms of semantics. A second limitation is related to the absence of procedures, such as calculating a competency index [39] to estimate the level of expert knowledge of the panel selected, for example. Nevertheless, the experts were believed to be trustworthy in their judgements because of their backgrounds and broad professional and academic experience. The third limitation refers to the fact that no analysis was made of the possible existence of differences between the sample of experts according to their sex. Nevertheless, it is considered that the panel of judges, selected for their theoretical and/or practical qualification, was, on the one hand, sufficiently heterogeneous in terms of their origin and type of qualification to collect different perspectives and alternatives of a problem or topic, thus providing a broad knowledge basis and producing high quality constructions; and on the other hand, it was also sufficiently homogeneous in terms of the level and degree of training, such that the number of experts that made up the aforementioned panel was methodologically pertinent to generate discussion and not to hinder the compilation and synthesis of information. A fourth limitation is related to the existence of possible differences between virtual versus semi-virtual work environments, and between private and public university institutions, which have not been verified.

Lastly, the qualitative nature of this study that facilitates the description and interpretation of several phenomena but not verification of them could be considered a limitation. This brings to the fore the need to do complementary studies that include a study perspective for men in order to contrast and generalize the results obtained for the reference population.

With respect to the practical implications, the study demonstrates the need to carry out prevention and training programs for university professors in psychosocial risks that emphasize characteristics particular to women at an organizational and personal level. At an organizational level, realistic changes to teaching loads and deadlines for delivering educational activities should be made. Working from home should be combined with working at the university to facilitate social contact and avoid isolation. Systematic institutionalized study plans should be mandated in which digital skills as well as knowledge related to the knowledge areas of the professors expertise are updated. Appropriate salary policies should be established and labor resources should be provided, such as psychological capital, which help cope with work related stressors and have a positive influence on work performance and psychosocial health [65]. Furthermore, taking actions at home workplaces to adapt them ergonomically and physically is a high priority. On the other hand, at a personal level, it is necessary to reinforce the personal resources of professors, such as working on emotional skills and encouraging healthy habits.

## Figures and Tables

**Table 1:** Sociodemographic characteristics of the experts.

Experts	*N*	%
	14	100%
Gender		
Male	2	14%
Female	12	86%
Field		
Academic	7	50%
Professional	7	50%
Qualification		
Professor	7	50%
OHST ^1^	10	71%
Years of experience		
1 to 5 years	1	7%
6 to 10 years	8	57%
over 11 years	5	36%
Age		
30 to 40 years	3	21%
40 to 50 years	9	64%
over 50 years	2	14%

^1^ Occupational Health and Safety Technician.

**Table 2:** Assignation percentage by experts for every risk factor.

Risk Factor	%	Risk Factor	%
Inadequate lighting	71.60	Electrical outlet overloads	35.71
Inadequate temperature	71.42	Social isolation	28.57
Noise level	64.29	Carpal tunnel syndrome	28.57
Static posture	64.29	Role ambiguity	28.57
Technostress	64.29	Not taking required breaks	28.57
Use VDT ^1^	57.14	Stress	28.57
Neck pain	57.14	Time pressure	28.57
Emotional overload	57.14	Quantity and complexity of inf.	28.57
Back pain	57.14	Non-ergonomic work equipment	28.57
Visual fatigue	57.14	Demands on attention	28.57
Impacts with objects	50.61	Lack of schedule	21.43
Falls on the same level or to a lower level	50.00	Sedentarism	21.43
Lack of autonomy	42.86	Difficulty with work–life balance	21.43
Fire	42.86	Sedentary position	21.43
Non-ergonomic table	42.86	Low social recognition	21.43
Non-ergonomic chair	42.86	Insufficient workspace	21.43
Headache	35.71	Level of humidity	21.43
Double presence	35.71	Burnout	21.43
Work spikes	35.71	Difficulties for promotion	14.29
Poor ventilation	35.71	Tobacco abuse	14.29
Job distance	35.71		

^1^ Visual Display Terminal.

**Table 3:** Comparison of frequency and percentage of agreement between experts and degree of consensus on each item.

Risk Factor	Frequency.	% Valid	Mean	Standard Deviation	Coefficient of Variation
Inadequate lighting	14	100	7.21	1.53	0.21
Inadequate temperature	13	92.9	6.62	2.02	0.30
Noise level	7	50	6.00	1.09	0.18
Static posture	14	100	8.07	1.82	0.22
Technostress	14	100	7.00	1.04	0.15
Used VDT	11	84.6	8.55	1.57	0.18
Neck pain	13	92.9	7.62	1.38	0.18
Emotional overload	14	100	6.29	1.27	0.20
Back pain	13	92.9	7.46	1.51	0.20
Visual fatigue	13	92.9	8.15	1.46	0.17
Impacts with objects	6	42.9	4.83	2.64	0.54
Falls on the same level or to a lower level	5	35.7	4.40	2.51	0.57
Lack of autonomy	9	64.3	6.78	1.48	0.21
Fire	6	42.9	3.33	1.63	0.48
Non-ergonomic table	11	78.6	6.91	1.58	0.22
Non-ergonomic chair	13	92.9	7.23	1.54	0.21
Headache	8	57.1	6.88	.99	0.14
Double presence	7	50	6.00	1.63	0.27
Work spikes	8	57.1	6.75	2.31	0.37
Poor ventilation	8	57.1	6.00	1.07	0.17
Job distance	4	28.6	6.75	1.71	0.25
Electrical outlet overloads	6	42.9	4.67	1.75	0.37
Social isolation	7	50	7.37	1.92	0.26
Carpal tunnel syndrome	9	64.3	6.89	1.83	0.26
Role ambiguity	9	64.3	6.44	1.33	0.20
Not taking required breaks	11	78.6	7.09	1.70	0.23
Stress	12	85.7	7.31	1.11	0.15
Time pressure	13	92.9	6.92	1.38	0.19
Quantity and complexity of information	9	64.3	6.22	1.56	0.25
Non-ergonomic work equipment	11	78.6	7.27	1.19	0.16
Demands on attention	10	71.4	6.20	1.62	0.26
Lack of schedule	10	71.4	6.50	2.27	0.34
Sedentarism	11	78.6	7.73	1.79	0.23
Difficulty with work–life balance	12	85.7	5.25	1.60	0.30
Sedentary position	10	71.4	7.10	1.66	0.23
Low social recognition	9	64.3	6.78	1.48	0.21
Insufficient workspace	10	71.4	6.30	1.64	0.26
Level of humidity	9	64.3	5.22	1.79	0.34
Burnout	9	64.3	6.89	1.54	0.22
Difficulties for promotion	7	50	7.14	1.34	0.18
Tobacco abuse	4	28.6	5.75	.96	0.16

**Table 4:** Risk factors and risks according to expert consensus.

Risks and Risk Factors	
Inadequate lighting	Back pain
Inadequate temperature	Visual fatigue
Static posture	Lack of autonomy
Technostress	Non-ergonomic table
Use VDT	Non-ergonomic chair
Neck pain	Carpal tunnel syndrome
Emotional overload	Demands on attention
Not taking required breaks	Stress
Role ambiguity	Time pressure
Non-ergonomic work equipment	Quantity and complexity of information
Lack of schedule	Sedentarism
Sedentary position	Low social recognition
Insufficient workspace	Burnout

**Table 5:** Final list of psychosocial risk factors according to expert consensus.

Consensus Psychosocial Risk Factors
Quantity and complexity of information
Demands on attention
Time pressure
Lack of schedule Role ambiguity
Emotional overload
Lack of autonomy
Not taking breaks
Low social recognition
